# Efficacy of MSC-derived small extracellular vesicles in treating type II diabetic cutaneous wounds: a systematic review and meta-analysis of animal models

**DOI:** 10.3389/fendo.2024.1375632

**Published:** 2024-07-15

**Authors:** Guangren Yue, Yu Li, Zheng Liu, Shuying Yu, Yilin Cao, Ximei Wang

**Affiliations:** ^1^ Plastic Surgery, The First Affiliated Hospital of Zhengzhou University, Zhengzhou University, Zhengzhou, China; ^2^ Department of Plastic and Reconstructive Surgery, Shanghai 9th People’s Hospital, Shanghai Jiao Tong University School of Medicine, Shanghai Key Laboratory of Tissue Engineering, National Tissue Engineering Center of China, Shanghai, China

**Keywords:** extracellular vesicle, exosome, mesenchymal stem cells, wound healing, animal models

## Abstract

**Background:**

Small extracellular vesicles derived from mesenchymal stem cells (MSC-sEVs) have emerged as a promising therapy for treating type II diabetic cutaneous wounds. Currently, the evidence supporting the use of MSC-sEVs for treating diabetic skin wounds remains inconclusive and is limited to preclinical studies. To facilitate the clinical translation of cell-free therapy, conducting a comprehensive systematic review of preclinical studies assessing the efficacy of MSC-sEVs is imperative.

**Methods:**

A systematic search was conducted on PubMed, Web of Science, Embase, and Cochrane Library databases until June 14, 2023, to identify studies that met our pre-established inclusion criteria. The outcome indicators comprised wound closure rate (primary outcome), neovascular density, re-epithelialization rate, collagen deposition, and inflammatory factors (secondary Outcomes). A fixed-effects model was employed in instances of low heterogeneity (I^2^<50%), while a random-effects model was utilized for high heterogeneity (I^2^≥50%). The risk of bias in animal studies was assessed using the SYRCLE tool.

**Results:**

Twenty-one studies were included in this meta-analysis. Compared with the control group, MSC-sEVs were found to significantly facilitate the healing of cutaneous wounds in type II diabetic patients (standardized mean difference [SMD]=3.16, 95% confidence interval [CI]: 2.65 to 3.66, P<0.00001, I^2 =^ 39%).

**Conclusions:**

According to the meta-analysis of preclinical studies, MSC-sEVs show promising applications in promoting type II diabetic wound healing. As a result, translating these findings into clinical applications appears warranted.

**Systematic review registration:**

https://www.crd.york.ac.uk/prospero, identifier CRD42023375467.

## Introduction

All previous meta-analyses failed to differentiate between animal models of type I diabetes mellitus (T1DM) and type II diabetes mellitus (T2DM). There are significant distinctions between T1DM and T2DM concerning their etiology, treatment approaches, and predisposing factors. This analysis preliminarily reveals the therapeutic effects of MSC-sEVs in T2DM animal models and provides a detailed evaluation of the credibility of these findings.

According to the International Diabetes Federation (IDF), the prevalence of diabetes is estimated to rise from approximately 10.5% in 2021 to around 12.2% by 2045 (1). Diabetes can be classified into several types based on their pathogenesis, such as T1DM, T2DM, gestational diabetes, and other types (2, 3). Specifically, T2DM stands out as the most predominant form of diabetes, making up approximately 90% of all diagnosed cases. Individuals with T2DM have a lifetime risk of approximately 30% of developing foot ulcers throughout their lives due to prolonged inflammation and weakened angiogenesis (4, 5). The presence of diabetic foot ulcers (DFUs) significantly impairs overall patient health and well-being, increasing their risk of mortality by 2.5 times within five years compared to individuals without DFUs (6). Regrettably, presently available clinical interventions for treating diabetic wounds are not universally effective, resulting in the need for amputations in around 15% of patients (6).

**Figure 1:**
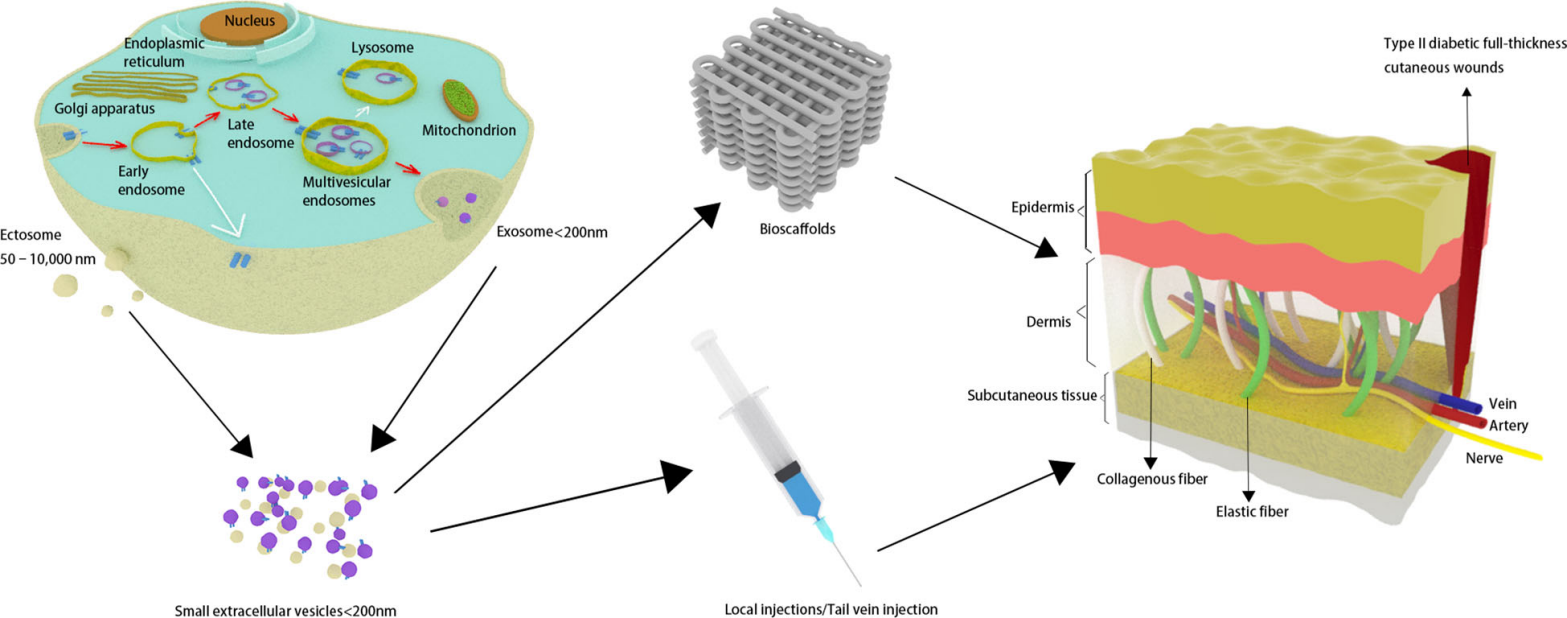
Small extracellular vesicles are secreted and have an effect on skin wounds in individuals with type 2 diabetes.

MSCs have recently emerged as an up-and-coming treatment option for various clinical diseases owing to their powerful regenerative capabilities and immunomodulatory properties. Despite the substantial capacity of MSCs to facilitate wound healing (7–9), challenges such as possible immunogenic rejection and chromosomal mutations hinder their direct transplantation applications (10). Meanwhile, it is worth noting that the regenerative effects of MSCs are mainly attributed to the secretion of extracellular vesicles (EVs) (11). Therefore, utilizing MSC-sEVs as a cell-free therapeutic approach holds considerable promise for overcoming the limitations inherent in MSC therapies (**Figure 1**).

EVs are secreted into the extracellular environment by diverse cell populations, encapsulated within a double-layered phospholipid membrane (12). The two primary types of EVs are exosomes, generated through the inward budding of the endosomal membrane, and ectosomes, released directly from the plasma membrane (13). Numerous mechanisms (including ESCRT machinery, the syntenin-Alix pathway, et al.) have been elucidated that regulate the biogenesis of EVs, intricately associating these processes with the molecular cargo of the EVs. These vesicles are enriched with a diverse array of biomolecules including proteins, long non-coding ribonucleic acids (lncRNAs), and microRNA molecules (miRNAs) (13). It is worth noting that exosomes are not vehicles of active DNA release, and they do not contain glycolytic enzymes or cytoskeletal proteins (14). Moreover, unlike the more direct formation of ectosomes, the biogenesis of exosomes necessitates the meticulously regulated transport of multivesicular endosomes (MVEs) to the plasma membrane. Most MVEs ultimately fuse with lysosomes, resulting in the degradation of their contents and thereby preventing the formation of exosomes. It is very difficult to completely separate exosomes from other types of EVs due to the limitations of current technologies used to isolate and detect EVs. EVs can be categorized based on their physical characteristics into “small extracellular vesicles” (sEVs) (<100 nm or <200 nm) and “medium/large extracellular vesicles” (>200 nm) (12). Thus, to mitigate potential inconsistencies and inaccuracies in nomenclature, it may be more advantageous to classify these structures as “small extracellular vesicles” instead of utilizing the terminology of “exosomes”. In this study, we performed a meta-analysis on MSC-sEVs smaller than 200 nm in diameter, including both exosomes derived from endosomes and ectosomes that sprout from the plasma membrane (15, 16).

Current research indicates that ADSC-sEVs hold significant potential as a therapeutic intervention for accelerating diabetic wound healing, primarily by promoting the regeneration of vasculature and cutaneous appendages (17, 18). It is worth noting that studies conducted to date suggest that sEVs demonstrate an acceptable safety profile without causing significant adverse reactions even upon repeated administration (19). These findings highlight the potential of MSC-sEVs as a viable treatment option for promoting diabetic wound healing. Despite the rapid advancement of therapeutic modalities utilizing MSC-sEVs, the evidence for application to treat type II diabetic wounds remains primarily preclinical and uncertain. This meta-analysis seeks to quantitatively assess the efficacy of MSC-sEVs in treating type II diabetic wounds. The findings from this comprehensive meta-analysis will provide valuable insights into the potential benefits and optimal use of MSC-sEVs as a promising therapeutic approach for type II diabetic wounds.

## Methods

This study registered on PROSPERO before starting (CRD2022327561). This meta-analysis follows the guidelines outlined in the Preferred Reporting Items for Systematic Reviews and Meta-Analysis (PRISMA) (20).

### Eligibility criteria

PICOS categories (Population; Intervention; Comparator; Outcome; Study design) were used to select trials for our systematic reviews and meta-analysis.

### Population

This study utilized animal models that developed T2DM either spontaneously or through the administration of streptozotocin (STZ) injections combined with a high-glucose and/or high-fat dietary regimen. We excluded studies involving only *in vitro*, or nonmammalian species (such as fish). Moreover, our investigation did not encompass scalding injuries arising from exposure to high temperatures or wounds resulting from radiation exposure. It is worth noting that our study excluded type I diabetes animal models.

### Intervention

To ensure a thorough analysis, our systematic review and meta-analysis encompassed all identified types of MSC-sEVs. Furthermore, the inclusion of sEVs obtained from fibroblasts in our analysis is warranted given the numerous resemblances between these cells and MSCs, including their capacity for adipogenic, osteogenic, and chondrogenic differentiation as well as similar profiles of cell surface markers (21, 22). Our article explores the use of xenogeneic, allogeneic, or autologous MSC-sEVs, which can be administered through various methods such as local subcutaneous injection, loading of sEVs into bioscaffolds, or intravenous injection. Our study examined the MSC-sEVs that were untreated, as well as those derived from parent cells subjected to preconditioning, such as hypoxia or overexpression of specific RNAs.

### Comparator

The control group was administered with nonfunctional solutions such as PBS or normal saline, bioscaffolds, or no treatment. Studies with missing experimental or control groups were excluded.

### Outcome

The primary outcome of this study was to determine the rate of closure of diabetic wounds, irrespective of their initial size or location (whether on the back or foot). The study also evaluated secondary outcomes, including vessel regeneration (measured by neovascular number and density), re-epithelialization, collagen deposition, and inflammatory response [measured by the expression levels of pro-inflammatory factors interleukin-6 (IL-6) and anti-inflammatory factor interleukin-10 (IL-10)]. To ensure the accuracy of our findings, we excluded *in vitro* experiments, clinical trials, and articles that lacked available data.

### Study design

All English-language, full-text, randomized controlled trials (RCTs) comparing MSC-sEVs with nonfunctional solutions (PBS or saline), bioscaffolds, or “no treatment” in type II diabetes wound animal models were included. Review articles, non-RCTs, commentaries, letters to the editor, care reports, case series, and repeated publications were excluded.

### Literature search strategy

As of June 14, 2023, a systematic search was conducted in PubMed, Web of Science, Embase, and Cochrane Library. See **Supplementary Table S1** for a specific search strategy. In addition, we conducted a manual search of research references to obtain potential studies.

### Study selection process

Endnote X9.3.3 was used to collect the articles found through a systematic search. Two independent reviewers screened the titles and abstracts of the articles and then screened the full text of any potentially relevant research. If there was any disagreement between the two reviewers, a third team member was consulted to achieve consensus through discussion.

### Data extraction

Two authors independently extracted relevant data extraction from screened articles, with any discrepancies settled through consensus with a third author. The following data were collected: (1) study characteristics (e.g., first author, year of publication, country of study); (2) study population (e.g., species, gender, body weight, method of diabetes model induction, wound size and location, etc.); (3) intervention characteristics (e.g., Minimal criteria for MSC identification of the International Society for Cell and Gene Therapy (ISCT), MSC source, MSC pretreatment method, gene overexpression and inhibition, dosage and administration method of MSC-sEVs, etc.); (4) study design (e.g., sample size, MSC-sEV isolation and characterization methods, etc.); (5) outcomes (e.g., wound closure rate, number and density of neovessels, collagen deposition, etc., and adverse events.

### Risk of bias

Two independent reviewers assessed the risk of bias in animal experiments using the Systematic Review Centre for Laboratory Animal Experimentation risk of bias (SYRCLE’s ROB) tool (23). Specifically, Selection bias: (1) sequence generation; (2) baseline characteristics; (3) allocation concealment; Performance bias: (4) random housing; (5) blinding of participants and personnel; Detection bias: (6) random outcome assessment; (7) Blinding of outcome assessment; Attrition bias: (8) incomplete outcome data; Reporting bias: (9) selective reporting; Other: (10) other sources of bias were assessed.

### Data analysis

Statistical analyses were conducted using Review Manager Software version 5.4 and STATA 17 (Stata Corp, College Station, TX, USA). All outcomes were classified as continuous data and presented as the SMD with 95% CIs, with a significant difference being P<0.05. As time points were inconsistent, the maximum effect estimate from each trial was utilized in the pooled analysis between 7 and 14 days. Heterogeneity between studies was assessed using the I^2^-statistic test: I^2^ ≤ 50% considered no significant heterogeneity, a fixed effects model was used, I^2^>50% considered significant heterogeneity, and a random effects model was used (24).

## Results

**Figure 2:**
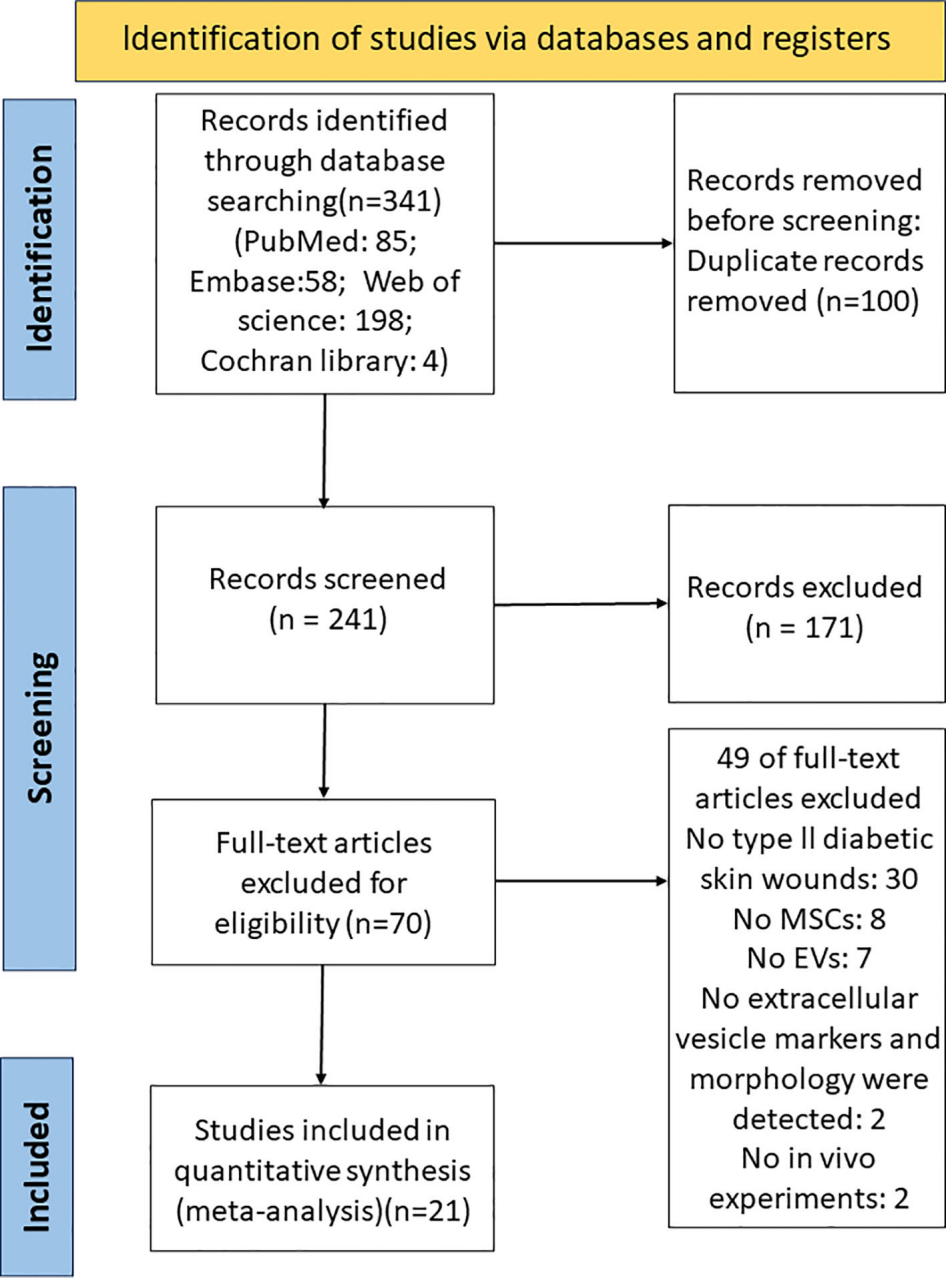
PRISMA flow diagram for the study selection process. PubMed, Web of Science, Embase, and Cochrane Library were searched from 2000 to June 14, 2023.

A total of 341 records were identified in the PubMed, Web of Science, Embase, and Cochrane Library databases before June 14, 2023. All articles were pooled into Endnote x9.3.3 software, and 100 duplicates were excluded. After screening titles and abstracts, 171 studies were excluded for focusing on non-diabetic skin wounds, *in vitro* studies, clinical studies, and other unrelated aspects. 70 studies were read in full, and 49 studies were excluded, of which 30 studies were not type II diabetic skin wounds, 8 studies isolated sEVs from cells other than MSCs, 7 studies did not address sEVs, 2 studies did not characterize sEVs by size, shape, and/or at least one sEV surface marker, and 2 studies were *in vitro* experiments. Finally, 21 studies were included in our meta-analysis (21, 25–44) (**Figure 2**).

### Characteristics of the included studies

From 2017 to 14 June 2023, a total of 21 eligible studies were included. Twelve articles (71%) were published from 2021 to 2023, reflecting the surge of interest in MSC-sEVs to promote type II diabetic skin wound healing in the last two years. The studies were from 3 different countries and regions. Nineteen studies were conducted in China, and one study was conducted in India and America.

Essential features of the animal study are summarized in **Table 1** Summary of animal study characteristics. All studies used rodents: rats (n=7) and mice (n=14). 57% of experiments (n=12) used male animals, 10% (n=2) used female animals, and 33% (n=7) did not report the sex of the animal model used. Notedly, it has been reported estrogen affects wound healing (45). In this subject paper, it was found that 8 studies utilized spontaneously diabetic mice, while the other 13 induced T2DM through the use of STZ and high-sugar and/or high-fat diets. The dose, timing, and route of STZ administration displayed heterogeneity across the examined investigations. It is worth noting that in two studies, STZ was injected via the tail vein (21, 39).

**Table 1:** Summary of animal study characteristics.

Study/(Location)	Animal model	Wound area/Position	Methods of inducing diabetes	Intervention(s), route and dose, and timing	Follow-up period	Comparator
**Han X. (** 21 **).**	Male 8 weeks SD rats (180–220 g)	Round full-thickness cutaneous wounds (2 cm diameter)/Back	Fed 45% high-fat diets for 5 weeks; STZ (35 mg/kg) via the tail vein	Injected subcutaneously with DF-Ex (2 mg in 200μL of PBS)	15 days	1) PBS 2) DF-Ex
**Li B. (** 25 **).**	Male 5 weeks C57BL/6J mice (20.88 ± 1.94 g)	Full-thickness cutaneous wounds (10 mm diameter)/On the dorsum hind feet	Fed with a high-glucose and high-fat diet for 6 weeks; intraperitoneally injected with 0.45% STZ(45 mg/kg)	Injected subcutaneously	13 days	1) PBS 2) MSC-Exo treated with oe-NC 3) MSC-Exo carrying on lncRNA H19
**Wang L. (** 26 **).**	Male 6-8 weeks SD Rats (200∼250 g)	Round full-thickness wound (1.5 cm diameter)/Back	Fed with a high-sugar and high-fat diet for 4 weeks; intraperitoneally injected with a single dose of 35 mg/kg STZ	Injected subcutaneously 100μg/ml BMSC-Exo (50μl*8)	14 days	1) PBS 2) BMSC-Exo 3) BMSC-Exo and Lenti-sh-Nrf2 4) BMSC-Exo and intravenous Lenti-sh-NC 5) BMSC-Exo and tert-butyl hydroquinone (TBHQ)
**Han Z. (** 27 **).**	Male BALB/C mice	Full-thickness cutaneous wounds (10×10 mm)/Back	Fed with a high-fat diet for 6 weeks; intraperitoneally injected with STZ (40 mg/kg, Sigma−Aldrich) for five consecutive days	100 µL of OE-NC-Exos, OE-KLF3-AS1-Exos, shNC-Exos or shKLF3-AS1Exos were delivered via tail vein injection	12 days	1) PBS 2) OE-NC-Exos 3) OE-KLF3-AS1-Exos 4) shNC-Exos 5) shKLF3-AS1-Exos
**Liang Z. (** 28 **).**	Male 5 weeks BALB/c mice (19.39 ± 1.56 g)	Full-thickness cutaneous wounds (10×10 mm)/Back	Fed with the high-fat diet; intraperitoneally injected with 0.45% STZ (45 mg/kg)	Exos derived from mmu_ circ_0001052-modified ADSCs or Exos from vector-modified ADSCs were subcutaneously injected. 25μL*4 of Exos (200 μg in 100 μL PBS) were subcutaneously injected	14 days	1) PBS 2) Exo+vector 3) Exos+mmu_circ_ 0001052
**Hsu. (** 29 **).**	10-12 weeks B6. Lepr db/db mice	Full-thickness cutaneous wounds (1 cm diameter)/Back	db/db mice	Repeated Exo administration was injected subcutaneously on days 1, 4, 7, 10, 13, and 16; 200 µg Exo suspended in PBS (200 µL)	17 days	1) PBS 2) ADSC-Exo
**Wang J. (** 30 **).**	4 weeks BALB/c nude mice	Full-thickness cutaneous wounds (0.8×0.8 cm)/Back	Fed with a 45-high-fat diet for 5 weeks; intraperitoneally injected with STZ (35 mg/kg)	ADSCs-Exo, HypADSCs-Exo (2 mg in 100 μL PBS), or 100 μL PBS were subcutaneously injected into four midpoints	14 days	1) PBS 2) ADSCs-Exo 3) HypADSCs-Exo
**Yan C. (** 31 **).**	Male 6 weeks C57BL/6J mice	Full-thickness cutaneous wounds (1.0×1.0 cm)/Back	Fed with a high-fat diet for 4 weeks; intraperitoneal administration of STZ(40 mg/kg	Injected subcutaneously with 100 µl of PBS; 50μg/ml of UC-MSC-Exos, and 100μg/ml of UC-MSC-Exos on days 0, 3, 5, 7, 9, and 11 after the establishment of the wound	14 days	1) PBS 2) 50μg/ml of UC-MSC-Exos 3) 100μg/ml of UC-MSC-Exos
**Zhao B. (** 32 **).**	Female 6 weeks db/db mice (36 to 42 g)	Full-thickness cutaneous wounds (1.5 cm^2^)/Back	db/db mice	No Treatment group and the other four groups applied PBS, rhEGF, and the cultmediumdium of hADSCs-CM or hADSCs-Ex three times a day at the wound site. A single application dose of hADSCs-Ex was 200 mg in 200 mL PBS	14 days	1) No Treatment group 2) PBS 3) rhEGF 4) hADSCs-CM 5) hADSCs-Ex
**Lv Q. (** 33 **).**	Male 5 weeks SD rats (150−200 g)	Full-thickness cutaneous wounds (15 mm diameter)/Back	Fed with a high-fat diet for 4 weeks; intraperitoneally injected with STZ at a dosage of 45 mg/kg	During wound treatment, 200 μL of solution with different therapeutic agents was evenly applied to the wound bed	15 days	1) PBS 2) miR-21 3) E-Exo (NC) 4) E-Exo
**Bian X. (** 34 **).**	Female diabetic mice (BKS-Dock Leprem2Cd479, db/db)	Full-thickness cutaneous wounds (16 mm diameter)/Back	db/db mice	dMSC-sEVs (100 μL, 5.22 × 10^11^particles/mL) and PBS (100 μL) were injected around the wounds at 4 sites (25 μL per site) at 7, 14, 21, and 28 days	28 days	1) PBS 2) dMSC-sEVs
**Shi Q. (** 35 **).**	Male SD rats (280–320 g)	Full-thickness cutaneous wounds (10 mm diameter)/Back	Fed with a high sucrose and high-fat diet for 10 weeks; intraperitoneally injected with STZ (35 mg/kg) at 10 and 11 weeks	100 µl PBS; hydrogel (13×13 mm) containing 100 µl PBS; hydrogel after loading 100 µl PBS containing 150 µg Exos. The wound dressings in each group were changed every 3 days	14 days	1) PBS 2) Chitosan/silk hydrogel 3) Chitosan/silk hydrogel +Exo
**Born. (** 36 **).**	db/db mice (40–50 g)	Full-thickness cutaneous wounds (12 mm diameter)/Back	db/db	50 µg (based on BCA protein content) of either HOTAIR-MSC-EVs, control transfected-MSC EVs, native MSC-EVs, or PBS as vehicle control, were administered around the wound four times in a cross pattern on day 3	14 days	1) PBS 2) MSC-EVs 3) Control transfected-MSC-EVs 4) HOTAIR-MSC-EVs
**Wei Q. (** 37 **).**	Male 8 weeks db/db mice	Full-thickness cutaneous wounds (10 mm diameter)/Back	db/db mice	UC-MSC-EVs (50 µL, 8.77×10^9^ particles/mL) were injected around the wounds at 4 sites (12.5 µL per site) every other day	12 days	1) 50 µL PBS 2) EV1: 1.84×10^9^/mL (150 µg/mL) 3) EV2: 2.04×10^9^/mL (167 µg/mL) 4) EV3: 2.25×10^9^/mL (184 µg/mL) 5) EV4: 2.45×10^9^/mL (200 µg/mL)
**Zhang Y. (** 38 **).**	7 weeks db/db mice	Round full-thickness cutaneous wounds (10 mm diameter)/Back	db/db mice	ADSC-Exos (200 µg) was injected by subcutaneous injection into the wound for three consecutive days	14 days	1) PBS 2) Exos
**Liu W. (** 39 **).**	Male SD rats (250-300 g)	Round full-thickness cutaneous wounds (10 mm diameter)/Back	Fed with a high-fat diet for 3 months; STZ (55 mg/kg) via caudal vein	Intradermal injected into the wound edge from four symmetrical directions on average through 29G needles. 200 μL PBS was injected in the same method as the control. For the inhibitor group, 200 μL PI3K-IN-1 (25 μg/mL) was coinjected with 200μL the ADSC-EVs in the same method.	14 days	1) PBS 2) ADSC-EVs 3) ADSC-EVs+ PI3K-IN-1
**Wang J. (** 40 **).**	4-5 weeks Nude BALB/c mice	Full-thickness cutaneous wounds (0.8×0.8 cm)/Back	A high-fat diet for 3–4 weeks; intraperitoneally injected with STZ (35 mg/kg)	EVs (2 mg in 100 μL PBS) or only 100 μL PBS subcutaneously at four points of the wound edge	14 days	1) Control 2) DM+PBS 3) DM+EVs 4) DM+ADSCs-hEVs 5) DM+ADSCs-cEVs
**Shiekh. (** 41 **).**	8 weeks Wistar rats	Full-thickness cutaneous wounds (8 mm diameter)/Back	Fed with a high-fat diet for 2 weeks; intraperitoneally injected with STZ (30 mg/kg); continuously fed with the high-fat diet for 4 weeks	PUAO, PUAO-CPO, PUAO-Exo, and PUAO-CPO-Exo scaffolds were applied on the wound beds, and the wounds were untreated in control groups; 100 μg/scaffold	14 days	1) Normal 2) PBS 3) PUAO 4) PUAO-CPO 5) PUAO-Exo 6) PUAO-CPO-EXO
**Liu Z.** (42)	Male 8 weeks db/db mice	Round full-thickness cutaneous wounds (1 cm diameter)/Back	db/db	Injected subcutaneously with 25 μL*4 of Exos (100 μg in 100 μL PBS)	14 days	1) PBS 2) Exo 3) PHE 4) PHE@Exo group
**Chen J.** (43)	8 weeks BALB/c mice	A square wound (1×1cm)/On the dorsal surface of the foot	Fed high glucose and high lipid diet; intraperitoneally injected with STZ (45mg/kg)	Injected subcutaneously (10 mg/kg in 100 μl PBS)	13 days	1) PBS 2) Exo 3) OE-circ-ITCH-Exo
**Zhao X.** (44)	Male 8 weeks C57BLKS-Leprdb (db/db) mice	Round full-thickness cutaneous wounds (1.5 cm diameter)/Back	db/db	Injected subcutaneously (20 μg in 100 μL PBS) every other day	21 days	1) PBS 2) Exo 3) Exo/eNOS

BMSCs, bone marrow mesenchymal stem cells; db/db mice, genetically modified diabetic mice; DFb, dermal fibroblast; DF-Ex, Exos derived from autologous dermal fibroblasts; DM, diabetes nude mice; dMSC-sEVs, sEVs derived from human decidua-derived mesenchymal stem cells; Exos, exosomes; Exo/eNOS, eNOS was substantially enriched in UCMSCs-exo; hADSCs-Ex, Exos from human adipose-derived mesenchymal stem cells; HOTAIR, HOX transcript antisense RNA; HypADSCs-Exo, hypoxic adipose stem cells Exos; lncRNA, long noncoding RNA; miR-21, miR-21-5p mimic; mixture, HA+PBS; NC, negative control; Nor, normal mice; Nrf2, transcription factor nuclear factor-E2-related factor 2; OE, overexpression; OE-circ-ITCH-Exo, overexpression of circ-ITCH or co-cultured with exosomal circ-ITCH; PHE, porous microspheres; PUAO, antioxidant polyurethane; PUAO-CPO, polyurethane-based oxygen-releasing antioxidant scaffolds; rhEGF, recombinant human epidermal growth factor; sh, silencing; SD, Sprague Dawley; SMSCs, synovium mesenchymal stem cells; STZ, streptozotocin; UC-MSC-EVs, umbilical cord-derived mesenchymal stem cells extracellular vesicles; USC-Exos, Exos from human urine-derived stem cells; USCsCon-shRNA-Exos, Exos from Con shRNA-transfected USCs; USCsshDMBT1 #1-Exos, Exos from DMBT1-silenced USCs.

Of the reviewed investigations, eighteen conducted local injections of sEVs into cutaneous wounds; one study implemented a tail vein injection method; while two studies utilized bioscaffolds laden with sEVs. However, only six studies specified the frequency of injection, which ranged from 1-6 times, and the timing of injection varied across all studies. The doses of MSC-EVs varied from approximately 50 μg to 2 mg (n=16) or 2.63*10^9^ to 5.22*10^10^ (n=2).

Essential characteristics of MSC-sEVs which are utilized in wound healing of animal models are summarized in **Table 2**. Although current procedures cannot identify the cellular origin of sEVs (46), a significant number of studies (16 out of 21) still describe them as “exosomes” of endosomal origin. It is concerning that more than half (12 out of 21) of the studies fulfilled the criteria for MSC proposed by the ISCT (47). The cells used in fifteen studies were derived from human tissues, while six were from mouse tissues. MSCs were isolated from various sources, including adipose tissue (n=10), bone marrow (n=4), umbilical cord (n=3), gingival tissue (n=2), dermal layer of skin (n=1), and decidua (n=1) (34).

**Table 2:** Summary of methods for isolation and characterization of MSC-sEVs.

Study/(Location)	Sources of sEVs	MSCs characteristics	Medium Supplementation	Isolation	sEVs characteristics		
					Size	Morphology	Markers
**Han X. (** 21 **).**	DFs from the skins of new-born rats	Not reported	Not reported	Centrifugation; ultracentrifugation; 0.22 μm filter; 100 kDa molecular weight cutoff (MWCO) hollow fiber membrane; washed 3 times with PBS	NTA: 100-120 nm	TEM: sphere-shaped	Positive: CD9, CD63 and ALIX; Negative: Calnexin
**Li B. (** 25 **).**	C57BL/6 mice BMSC	Chondrogenic, adipogenic, and osteogenic/CD73(100%), CD90(94.2%), CD105(97.8%), the rest(less than 1%)	Serum free DMEM	Ultracentrifugation	TEM; DLS: 30-120 nm	TEM: round or oval	Positive: CD63, CD81, TSG101 and heat shock protein 70 (HSP70) Negative: GRP94
**Wang L. (** 26 **).**	SD rats BMSC	CD105(97.3%), CD90(95.8%), and CD45(0.33%)	Not reported	Centrifugation; ultracentrifugation	TEM: around 100 nm	TEM: spherical double-membrane	Positive: CD9, CD63 and TSG101
**Han Z. (** 27 **).**	BMSC	Positive: CD29, CD44, and CD106/Negative: CD34 and CD45	Serum-free DMEM	Centrifugation; ultracentrifugation; 0.22 μm sterilized filter	NanoSight NS500 instrument: 50-150 nm	TEM (shape not described)	Positive: CD63, TSG101, and CD9; Negative: GM130 (Golgi apparatus marker) and calnexin (an endoplasmic reticulum marker)
**Liang Z. (** 28 **).**	BALB/c mice ADSC	Chondrogenic, adipogenic and osteogenic/CD73(99.87%), CD90(99.91%), CD105(99.84%), and CD34(5.56%)	FBS-free endothelial cell growth medium (EGM)-2MV	Differential ultracentrifugation	TEM: 30-200 nm	TEM: round shape, saucer shape, or hemispherical double-layer pattern structure with one-sided depression	Positive: CD63 and CD81
**Hsu. (** 29 **).**	db/db mice ASC and DFb	Not reported	5% Exo depleted FBS	Centrifugation; Exo-spin Exo purification kit	NanoSight: ASC-Exo: 119.1 ± 27.2 nm DFb-Exo: 110.2 ± 23.5 nm	TEM: doubled layered cup-shaped	Positive: CD9, CD63 and CD81
**Wang J. (** 30 **).**	ADSC	Not reported	Microvascular endothelial cell growth medium‐2 media deprived of FBS	Centrifugation; 0.22 µm sterile filter; ExoQuick‐TC reagent	TEM: 110 nm	TEM: round	Positive: HSP70 and CD9
**Yan C. (** 31 **).**	UCMSC	Not reported	RPMI 1640 (Sigma-Aldrich, USA, cat. no. R8758), containing 10% exosome-depleted FBS	Ultracentrifugation; 0.2 µm filter; washed with PBS	DLS: 30-150 nm	TEM: homogeneous, spherical, and membrane	Positive: CD9, CD81 and tumor susceptibility gene 101 (TSG101)
**Zhao B. (** 32 **).**	ADSC	Adipogenic, osteogenic and chondrogenic/Positive: CD73 CD90 and CD105; Negative: CD45 and HLA-DR	Not reported	Not reported	Not reported	Not reported	Positive: CD9, CD63 and CD81
**Lv Q. (** 33 **).**	ADSC	Not reported	FBS-free medium	Centrifugation; ultracentrifugation; 0.22 μm filter; 100 kDa ultrafiltration unit	NTA: 41-130 nm	TEM: cup and sphere	Positive: CD63, CD9, and TSG101
**Bian X. (** 34 **).**	hdMSC	Osteogenic, adipogenic, and chondrogenic/CD90 (97.33%) CD73 (98.11%) CD105 (99.97%) CD19 (0.10%) CD45 (0.20%) CD34 (0.20%) HLA-DR (0.00%)	DMEM/F12 containing 10% Exo-free FBS	Centrifugation; ultracentrifugation; washed with PBS	NTA: 63.8-125 nm; TEM	TEM: cup	Positive: CD9, CD63, CD81, and TSG101 Negative: Grp94
**Shi Q. (** 35 **).**	GMSCs	Osteogenic, adipogenic and chondrogenic/CD44 (99.99%) CD73 (100%) CD90 (99.87%) CD105 (100%) and CD29 (99.53%) CD31 (0.75%) CD34 (0.17%) and CD45 (0.54%)	DMEM/F12 supplemented with 10% Exo-free FBS	Centrifugation; 0.22 µm filter; 30 kDa molecular weight cutoff (MWCO) hollow fiber membrane; qEV column and was eluted with PBS	TRPS analysis: 127 ± 55.9 nm	TEM: spherical	Positive: CD9 and CD81
**Born. (** 36 **).**	BMSC	Not reported	EV-depleted FBS media	Centrifugation; ultracentrifugation; washed with PBS; Nanosep centrifugal devices with 300 kDa MWCO mega Membranes	NTA: 100–120 nm	TEM	Positive: TSG101 and CD63 Negative: calnexin and serum transport protein albumin
**Wei Q. (** 37 **).**	UCMSC	Chondrogenic, adipogenic, and osteogenic	DMEM/F12 containing 10% EV-free FBS	Centrifugation; ultracentrifugation; 0.22 μm sterile filter	NTA: 60-180 nm (mean diameter 100 nm)	TEM: saucer	Positive: TSG101, CD63 and CD9
**Zhang Y. (** 38 **).**	ADSC	Adipogenic and osteogenic/Positive: CD90 and CD105 Negative: CD31 and CD34	Exo-free 10% (v/v) FBS (Exos were depleted by ultracentrifugation for 16h at 120,000g)	Centrifugation; ultracentrifugation	Not reported	TEM: bilayer cup-shaped	Positive: CD63 and CD9
**Liu W. (** 39 **).**	BMSC	Adipogenic, osteogenic, and chondrogenic/Positive: CD105 CD90 CD73; Negative: CD45 and CD34	Serum-free culture medium	Centrifugation; ultracentrifugation; 0.22 μm filter	TEM: approximately 120 nm Nanosight: 30-150 nm	TEM: oval bilayer lipid membrane	Positive: CD81, Tsg101, Alix; Negative: Calnexin
**Wang J. (** 40 **).**	ADSC	Adipogenic and osteogenic/Positive: D44 CD29 and CD105 Negative CD14 and CD34	10% serum without EVs	Centrifugation; 0.22 μm sterile filter; ExoQuick-TC reagent	NTA: a mean size of 110 nm	TEM	Positive: CD63, TSG101, and CD9
**Shiekh. (** 41 **).**	Rats ADSC	Not reported	10% Exo-free FBS	Centrifugation; ultracentrifugation; 100 kDa filter; washed with Milli-Q water	NanoSight system: the average size of 165.7 nm	TEM, SEM: cup-shaped	Positive: CD81; Negative: β-actin
**Liu Z. (** 42 **).**	GMSCs	Chondrogenic, adipogenic, and osteogenic/Positive: CD44, CD90, and CD105; Negative: CD34 and CD45	DMEM/F12 containing 10% exosome-free FBS	Centrifugation; ultracentrifugation;	NTA: 135.2 ± 44.3 nm	TEM: spherical structure	Positive: CD63 and Tsg101
**Chen J. (** 43 **).**	BMSCs	Not reported	Not reported	Centrifugation; ExoQuick-TC solution	NTA: 50-150 nm	TEM: round or oval membranous vesicles	TSG101, Alix, CD9, CD63, and CD81
**Zhao X. (** 44 **).**	UCMSCs	Chondrogenic, adipogenic, and osteogenic/Positive: CD90, CD105, and CD73; Negative: CD45, CD34, CD14, CD11b, CD19, and HLA-DR	Exosome-depleted medium	Centrifugation; ultracentrifugation; 0.22 µm flter	DLS: around 78.82 nm	TEM: bilayer membrane structure	TSG101, CD9, CD63, CD81

ADSC, adipose-derived stem cell; AMSC, adipose-derived mesenchymal stem cell; BCA, bicinchoninic acid; BMSC, bone marrow mesenchymal stem cell; DFs, dermal fibroblasts; DLS, Dynamic Light Scattering; FBS, fetal bovine serum; GMSC, gingival mesenchymal stem cell; hdMSC, human decidua-derived mesenchymal stem cell; NTA, nanoparticle tracking analysis; SEM, scanning electron microscope; TRPS, tunable resistive pulse sensing; TSG101, tumor susceptibility gene 101; UCMSC, umbilical cord-derived mesenchymal stem cell; USC, urine-derived stem cell.

In eighteen studies, sEVs-depleted serum medium was used. However, only one article reported the depletion of exosomes through ultracentrifugation for 16 hours at 120,000 g and the other three studies did not mention the use of sEVs-depleted serum. Out of the total studies reviewed, most (n=19) described the enrichment process of sEVs in detail. One study mentioned the preparation of sEVs through “differential ultracentrifugation”, while another did not mention it at all. Fifteen studies utilized ultracentrifugation to isolate sEVs, with eight of them combining filtration. Three studies used ExoQuick, a reagent for exosome isolation, while one study used the Exo-spin Exo purification kit, and another used a qEV column. Furthermore, several emerging methods for sEV isolation have been developed, including field-flow fractionation (FFF), asymmetric flow field-flow fractionation (AFFF, A4F, or AF4), and field-free viscoelastic flow.

In this analysis, all articles tested sEVs using at least one of three methods: morphology, size, and surface markers. The size was determined using several techniques, including nanoparticle tracking analysis (NTA) (n=9), transmission electron microscopy (TEM) (n=3), dynamic light scattering (DLS) (n=3), tunable resistant pulse sensing (TRPS) (n=1), a combination of two methods (n=3), and two studies did not detect. The morphology of sEVs was observed by TEM (n=19). In one study, the method of visualization was not mentioned, while another study utilized both TEM and scanning electron microscope (SEM). The sEVs observed in these studies displayed a characteristic spherical or cup-shaped morphology. Out of the fourteen studies, protein quantification was carried out using the bicinchoninic acid assay (BCA). Only one study utilized the A280 (nm) UV absorption method with a NanoDrop One (Thermo Fisher Scientific), whereas six studies did not mention the method of detecting protein quantification. The specific surface markers of sEVs were detected by using Western blotting (n=18), flow cytometry (n=1), and both methods (n=2).

### Primary outcome–wound closure

**Figure 3:**
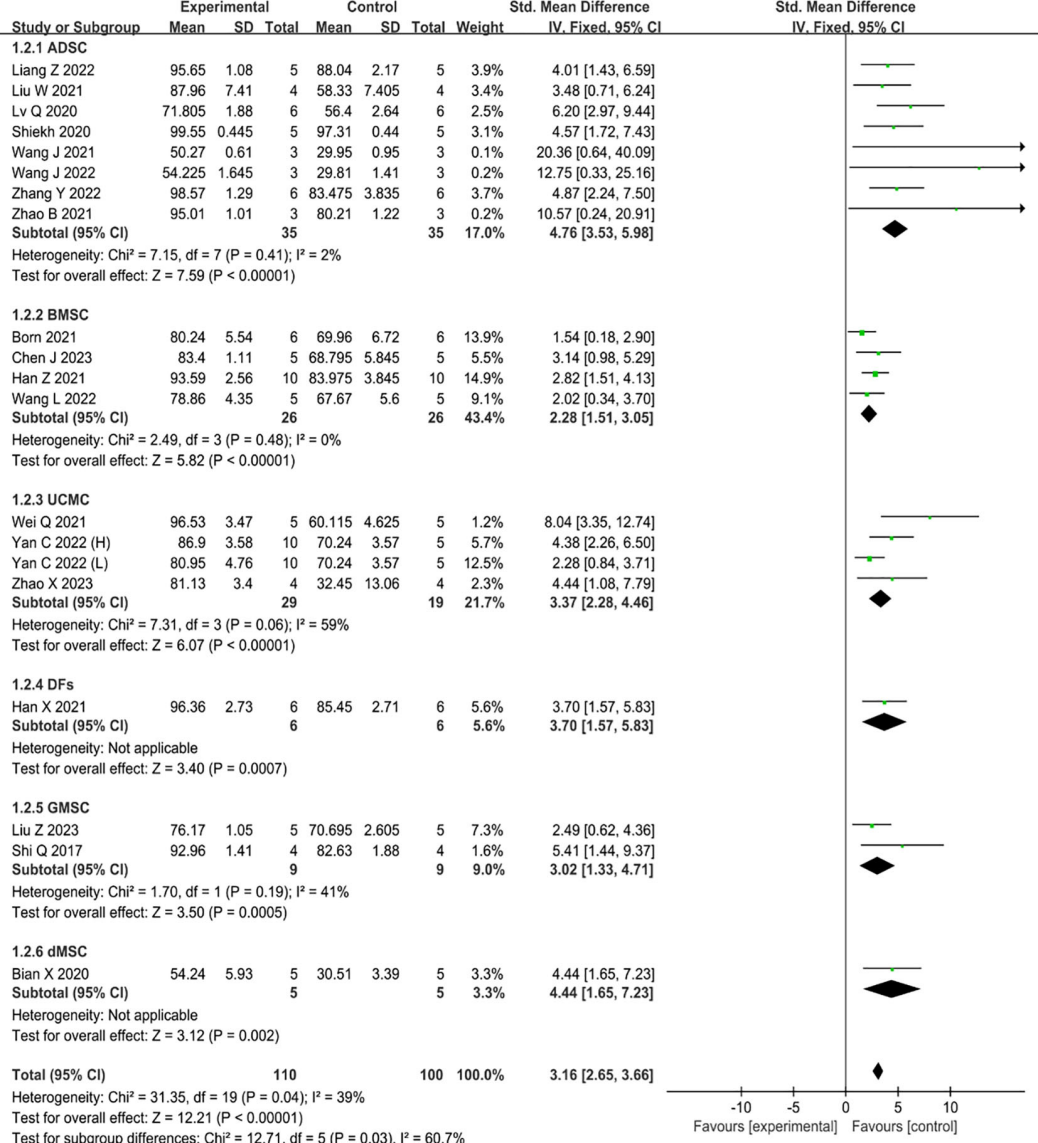
Forest plot comparing wound healing in type II diabetic wound models after intervention using MSC-sEVs compared to controls. ‘Yan C 2022 (H)’ represents a higher concentration, while ‘Yan C 2022 (L)’ represents a lower concentration.

Nineteen studies reported data on wound closure, one of which compared the therapeutic benefits of two concentrations of UCMC-sEVs (31). The results of these studies revealed that the incorporation of MSC-sEVs expedited the healing process of diabetic skin wounds significantly when compared to the control group (SMD=3.16, 95% CI: 2.65 to 3.66, P<0.00001, I^2^ = 39%) (**Figure 3**). Notably, a significant decrease in heterogeneity was observed in the subgroup analysis between the ADSC (I²=2%) and BMSC (I²=0%) groups, suggesting that the heterogeneity of the findings may be related to the source of sEVs.

### Secondary outcomes

#### Angiogenesis

**Figure 4:**
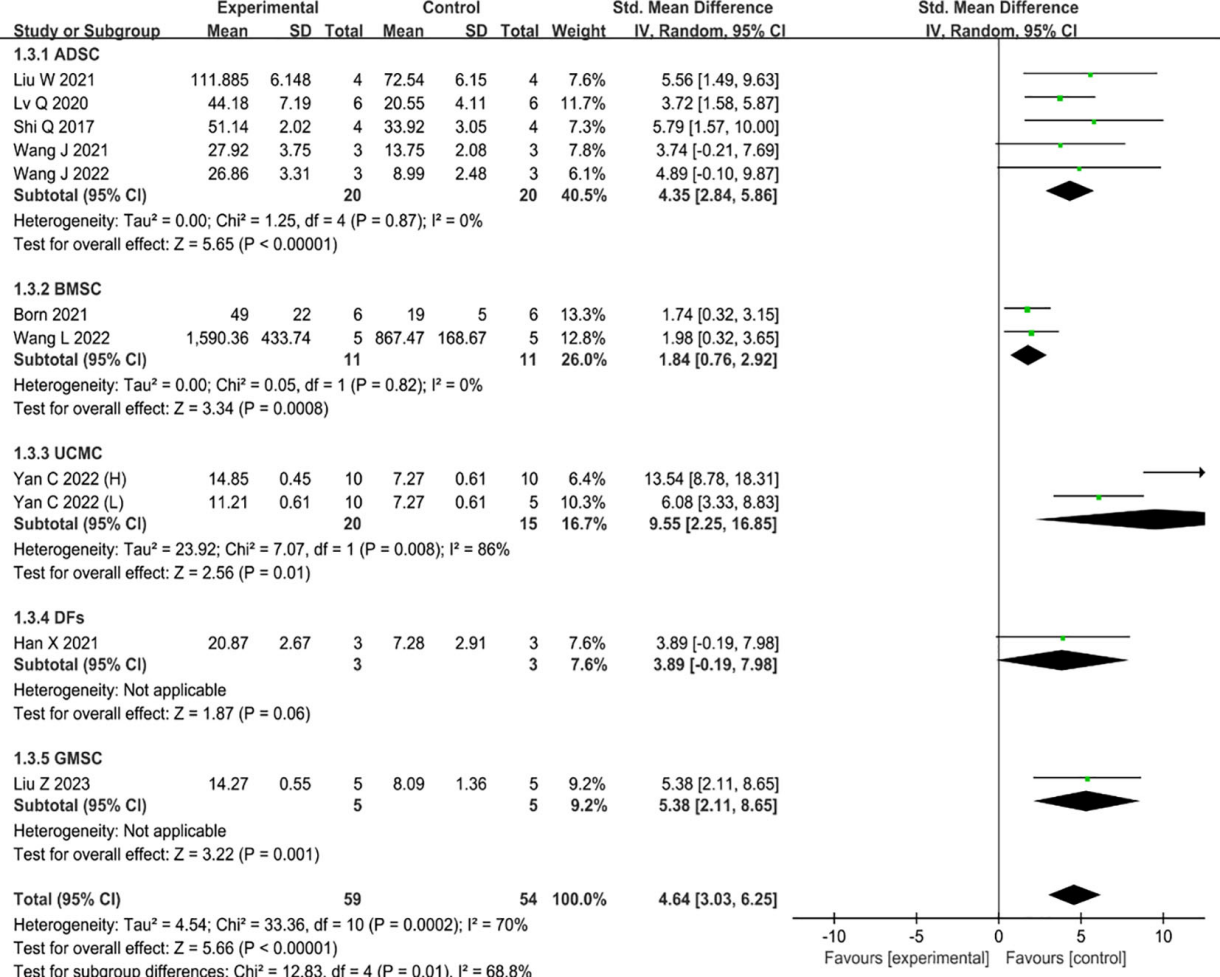
Forest plot comparing the revascularization of a diabetic wound model after intervention using MSC-sEVs compared to controls.

Ten studies reported data on the promotion of revascularization by MSC-sEVs, one of which compared the therapeutic benefits of two concentrations of UCMC-sEVs (30). The results of these studies have shown that the inclusion of MSC-sEVs in type II diabetic skin wounds significantly enhances revascularization when compared to the control group (SMD=4.64, 95% CI: 3.03 to 6.25, P<0.0001) (**Figure 4**). However, there was high heterogeneity (I^2^ = 72%). Consistent with the above conclusion, subgroup analysis suggests that different types of parental cells may contribute to increased heterogeneity (**Figure 4**).

#### Re-epithelialization

**Figure 5:**
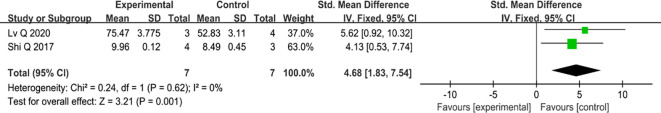
The forest map compares the re-epithelization of the diabetes wound model after MSC-sEV intervention with that of the control group.

Only two studies have examined the re-epithelialization of type II diabetic wounds, and both found positive results. When compared to the control group, the addition of MSC-sEVs significantly accelerated the re-epithelialization process of type II diabetic skin lesions (SMD=4.68, 95% Cl: 1.83 to 7.54, P=0.001). Furthermore, the heterogeneity test showed that there was no significant heterogeneity (I^2^ = 0%) (**Figure 5**).

#### Collagen deposition

**Figure 6:**
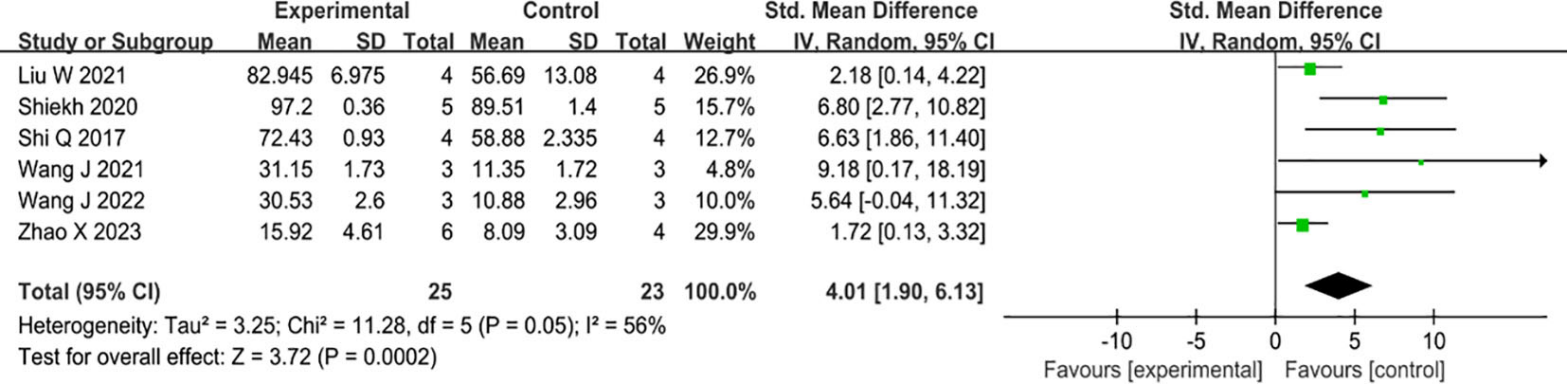
Forest plot comparing total collagen deposition in diabetic wound models after intervention using MSC-sEVs compared to controls.

Six studies have examined the effects of MSC-EVs on collagen deposition in type II diabetic animal wound models, all of which have reported positive outcomes. The administration of MSC-sEVs markedly enhanced collagen fiber deposition compared to the control group. (SMD=4.01, 95% Cl: 1.90 to 6.13, P<0.00001). The heterogeneity test showed that there was significant variation among the studies (I^2^ = 56%) (**Figure 6**).

#### Inflammation

**Figure 7:**
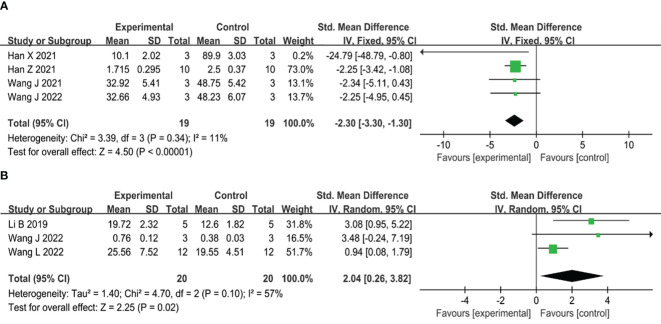
**(A)** Forest plot comparing inflammatory IL-6 expression in type II diabetic wound models following intervention using MSC-sEVs compared to controls. **(B)** Forest plot comparing anti-inflammatory factor IL-10 expression in type II diabetic wound models following intervention using MSC-sEVs compared to controls.

Six studies have shown that the use of MSC-sEVs can effectively reduce inflammation in type II diabetic wounds. Out of the six studies, four reported administration of MSC-sEVs significantly reduced the expression levels of IL-6 compared to controls (SMD=-2.30, 95% CI: -3.30 to -1.30, P<0.00001), which showed no significant heterogeneity (I^2^ = 11%) (**Figure 7A**). On the other hand, three studies reported administration of MSC-sEVs significantly elevated the expression the levels of IL-10 compared to controls (SMD=2.04, 95% CI: 0.26 to 3.82, P=0.02), an anti-inflammatory cytokine, but the significant heterogeneity was observed (I^2^ = 57%) (**Figure 7B**).

#### Risk of bias and publication bias of included studies

**Figure 8:**
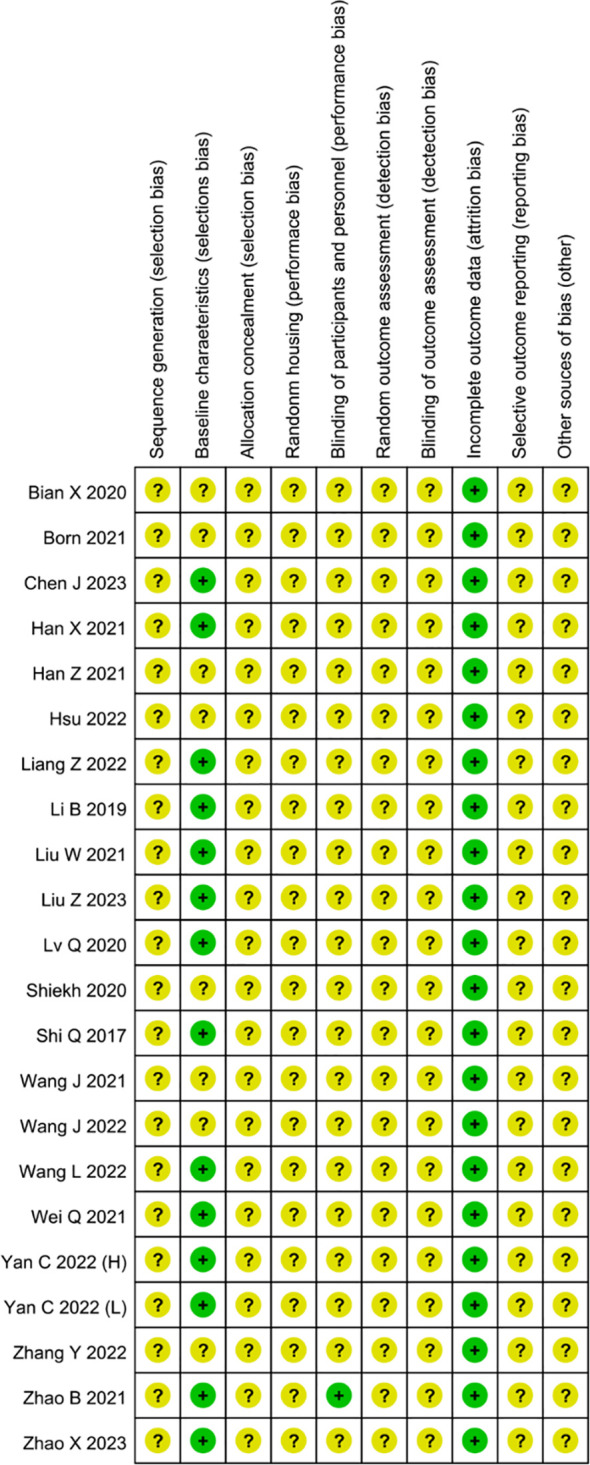
Risk of bias assessment of the 18 reviewed studies based on SYRCLE’s ROB tool represented by RevMan 5.4.1.

In our meta-analysis, all studies across most domains were assessed to have an unclear risk of bias (**Figure 8**). Thus, the overall risk of bias in all trials was deemed unclear. In all trials, the risk of bias associated with random sequence generation was estimated as unclear, due to the lack of detailed descriptions of the randomization method, such as the random number method. Twelve studies (21, 24–26, 28, 31–33, 35, 37, 38, 42) reported comparable baseline characteristics, whereas nine studies could not make judgments due to insufficient reporting of individual characteristics, particularly age (27, 34, 36) and sex (29, 30, 36, 39–41, 43). None of the publications explicitly provided information on allocation concealment; consequently, all studies were deemed to have an unclear risk of bias in this domain. Random housing was deemed unclear risk of bias across all studies, as none provided detailed descriptions of procedures for randomly assigning animals in animal rooms. In one trial, participants were blinded to the intervention, as it explicitly stated that all animal experiments were conducted by an investigator blinded to the drug treatments (32). Consequently, we assessed it as having a low risk of bias for blinding of participants and personnel whether other studies were assessed as unclear. In all studies, the risk of bias for random outcome assessment was deemed unclear, as no study provided detailed descriptions for the random selection of animals for outcome assessment. Blinding of outcome assessment was at unclear risk of bias in all experiments, attributable to the absence of measures ensuring outcome assessors were blinded to the interventions administered to the animals. No article indicated whether attrition and exclusions were reported, contributing to incomplete outcome data being rated as an unclear risk of bias. We assigned an unclear risk for selective outcome reporting to all trials, as insufficient information was available to make a definitive judgment. No additional sources of bias were identified, and all studies in this field were rated at low risk of bias.

We inspected the symmetry of the funnel plot when sufficient studies (more than 10) were included in a forest plot (Wound Closure). Our findings revealed a symmetrical funnel plot, indicating significant evidence of publication bias in our study (**Supplementary Figure S1**). The “Egger test” was performed to discern the presence of publication bias, providing empirical evidence that supports the existence of such bias (p=0.000) (**Supplementary Table S2**). Consequently, trim-and-fill was employed to evaluate the potential influence of publication bias on the initial conclusion. Through this analysis, a total of 9 theoretically missing experiments, were denoted by boxed circles (**Supplementary Figure S1**). The recalculated summary analysis results demonstrate sustained consistency without any instances of reversal, thereby indicating the robustness and stability of our analysis findings (pre-trim-and-fill: standardized mean difference [SMD] = 2.12, confidence interval [CI] = 2.649-3.662, p = 0.000; post-trim-and-fill: SMD = 5.39, CI=9.512-24.381, p=0.000) (**Supplementary Table S3**).

## Discussion

Our systematic review and meta-analysis evaluated the therapeutic efficacy of MSC-sEVs in preclinical models of diabetic skin wounds. The administration of MSC-sEVs significantly improved diabetic wound closure by promoting vascular regeneration, enhancing collagen synthesis, and modulating inflammatory responses. Some studies have demonstrated that ADSC-sEVs exhibit a more pronounced effect on promoting vascular regeneration than BMSC-sEVs (48–50). ADSC-derived sEVs have demonstrated superior efficacy in promoting diabetic wound healing (50). Moreover, gene and protein analyses have indicated that ADSC-sEVs contain higher levels of VEGF (48, 49). However, the results from multiple subgroup analyses in our meta-analysis may introduce complexity and potential for misinterpretation, thereby preventing clear conclusions from being drawn. It is worth noting that subgroup analysis revealed that the source of sEVs could be a significant factor contributing to the observed high heterogeneity in the analysis (51).

Our meta-analysis revealed that local injection was the predominant route of administration for MSC-sEVs (18/21). Although MSC-sEVs hold promise for regenerative therapy, their rapid systemic clearance presents a significant challenge (52, 53). Studies have demonstrated that administering multiple doses of MSC-sEVs leads to superior regenerative efficacy compared with a single equivalent dose (54). Moreover, utilizing novel materials in conjunction with MSC-sEVs offers the potential to achieve sustained extracellular vesicle release. Administration of MSC-sEV-loaded OxOBand wound dressings, a highly porous cryogel, has demonstrated the capacity to reduce oxidative stress levels while promoting wound healing among individuals diagnosed with T2DM (41).

Current research indicates that enhancing the therapeutic potential of sEVs may be possible by preconditioning MSCs or upregulating expression levels of certain types of RNA. Hypoxia preconditioning of ADSCs has been demonstrated to enhance the therapeutic efficacy of sEVs derived from these cells, chiefly attributed to their facilitation of fibroblast proliferation and migration (30). Moreover, enrichment of long non-coding RNA H19 (lncRNA H19) in MSC-sEVs has been found to facilitate fibroblast proliferation and migration by activating the phosphatidylinositol-4,5-bisphosphate 3-kinase/protein kinase B (PI3K/Akt1) signaling pathway (28). The overexpression of KLF3-AS1, an exosomal lncRNA derived from BMSCs, resulted in the downregulation of miR-383 and upregulation of its target gene VEGFA (26). Electroporation was used to load miR-21-5p mimics into sEVs, which stimulated the proliferation and migration of keratinocytes through the Wnt/β-catenin signaling pathway (33). Despite extensive research that has been undertaken on sEVs, several fundamental questions are still unanswered. Specifically, there remains a lack of clarity regarding the mechanisms by which these structures transport their contents to host cells and the specific molecular pathways underlying their diverse physiological impacts.

Inadequate vascularization is a crucial factor contributing to the non-healing of skin wounds in individuals with T2DM (55). In numerous studies, researchers commonly employ the ratio of CD31 and α-SMA positive cells as an indicator to evaluate angiogenesis in the wound bed of patients diagnosed with T2DM. MSC-sEVs play a vital role in promoting angiogenesis by inducing the expression of vascular endothelial growth factor (VEGF) as well as stimulating endothelial cell proliferation and migration via the PI3K/AKT pathway (56). ADSC-sEVs upregulate HIF-1α and VEGF expression through the PI3K/AKT signaling pathway, thereby promoting wound angiogenesis in individuals with T2DM (39). Additionally, emerging evidence suggests that extracellular RNA (exRNA) secreted by MSC-sEVs plays a significant function in the PI3K/AKT signaling pathway (13). By specifically targeting the PTEN/AKT/HIF-1α/VEGF signaling pathways, miR-17-5p, found within UCMSC-sEVs, facilitates the activation of endothelial cells (37).

Impaired macrophage function constitutes a significant etiologic factor underlying the chronic inflammation observed in individuals with T2DM (57–59). The outcomes from our meta-analysis clearly demonstrate that treatment with MSC-sEVs results in a notable reduction in IL-6, a prominent proinflammatory factor, while concomitantly elevating the expression of IL-10, a well-established anti-inflammatory marker. UCMC-sEVs have been demonstrated to promote wound healing through the alleviation of oxidative stress-mediated injury at the site of tissue damage (31). An excessive number of proinflammatory M1 macrophages at sites of tissue damage in patients afflicted by T2DM constitutes a major etiological factor underlying persistent inflammation on the skin wound surface (60). Additionally, the phenotypic transformation of macrophages from a proinflammatory M1 to an anti-inflammatory M2 state constitutes a pivotal factor influencing various cellular functions such as proliferation, motility, and vascularization (60). Recent investigations have suggested that ADSC-sEVs can shift macrophages toward an anti-inflammatory M2-like phenotype and hypoxic preconditioning elicits further enhancements in their therapeutic potential (61). Notably, prolonged stimulation of M2-like macrophages can trigger exaggerated synthesis and deposition of collagen in the extracellular matrix leading to abnormal tissue scarring (62).

Our systematic review and meta-analysis revealed that MSC-sEVs are capable of augmenting the process of collagen deposition at sites of cutaneous injury among individuals diagnosed with T2DM. Treatment with ADSC-sEVs was found to enhance the regeneration of collagen by suppressing the expression of matrix metalloproteinase 1 (MMP1) and matrix metalloproteinase 3 (MMP3) (31). It should be noted that although the deposition of type I and III collagen plays a vital role in the process of wound healing, an excessive amount may result in adverse outcomes such as keloid formation (63). Most studies had a follow-up duration of less than 21 days post-wounding, which greatly limited their ability to comprehensively evaluate collagen growth and maturation in healing tissues (64).

Although sEVs have been recognized as a promising therapeutic approach, their clinical application still faces numerous challenges. For clinical applications, it is of paramount importance to ensure the stability and safety of treatment outcomes. However, the current production process of sEVs encompasses numerous variables, such as cell source, separation method, and cell culture conditions, all of which can impact the final yield and composition of sEVs. Furthermore, the mass production of sEVs remains a critical issue that requires urgent attention. Certain experimental approaches have achieved significant yields of sEVs through large-scale cell culture techniques, such as platform rocker wave bags (up to 500 L scale), to obtain substantial quantities of cell supernatant (65). Bacterial contamination can be effectively eliminated throughout the production process through sterile filtration; however, viral contaminants can easily co-purify with sEVs, posing significant medical safety concerns. Therefore, developing an effective detection method for sEVs products is imperative, as this represents a key challenge in the clinical translation of sEVs-based therapies. However, the absence of standardized assays has resulted in each laboratory and company employing disparate testing methods tailored to their specific sEV-based therapeutics. Overall, the selection of parental cells, the cell culture process, and the detection of sEVs-related therapeutics can all significantly impact the safety of patient treatments. However, no regulatory agency has yet issued comprehensive guidelines for testing the safety and efficacy of these sEVs. Overall, despite the absence of a standardized protocol for MSC-sEV extraction, current preclinical studies have demonstrated the promising therapeutic potential of these vesicles.

There are still several noteworthy limitations to our study. First, according to the Sycle RoB tool, the bias risk analysis conducted in this systematic review indicates that the overall risk of bias across all studies is rated as unclear. In preclinical research, the reporting of methodological details *in vivo* studies is generally inadequate. Notably, none of the studies included in our meta-analysis indicated compliance with the ARRIVE guidelines (Animal Research: Reporting of *In Vivo* Experiments) (66). Therefore, in our systematic review, the data obtained may be subject to overestimation or underestimation, thereby potentially compromising the reliability of research conclusions and limiting their clinical translational significance (67). As a result, there is an urgent need to enhance adherence to the ARRIVE guidelines to effectively address the predominantly unclear risk of bias in animal research. In this meta-analysis, owing to the absence of mandatory reporting standards for preclinical animal studies, as well as feasible power calculations and research protocols, studies will not be excluded based on low-quality ratings. Second, owing to the inherent limitations of preclinical disease models, murine models might not comprehensively replicate every physiological alteration that occurs in individuals with type II diabetes. Third, several investigations failed to meet the minimal consensus criteria for MSCs proposed by ISCT. Fourth, it is important to acknowledge that the meta-analysis results may have been influenced by the varying injection concentrations and frequencies of MSC-sEVs utilized across different studies.

## Conclusion

To summarize our findings, MSC-sEVs demonstrated expedited wound healing properties in cutaneous injuries among T2DM animal models. Importantly, we identified inherent risks of bias s throughout all enrolled investigations that constrained the breadth and generalizability of our findings. Consequently, we advocate for the implementation of an extensive randomized controlled trial at a larger level and with prolonged follow-up durations to corroborate these preliminary findings.

## Data availability statement

The datasets presented in this study can be found in online repositories. The names of the repository/repositories and accession number(s) can be found in the article/**Supplementary Material**.

## Author contributions

GY: Data curation, Investigation, Methodology, Resources, Software, Supervision, Validation, Writing – original draft, Writing – review & editing. YL: Conceptualization, Data curation, Formal analysis, Project administration, Visualization, Writing – original draft, Writing – review & editing. ZL: Formal analysis, Investigation, Methodology, Visualization, Writing – review & editing. SY: Conceptualization, Methodology, Validation, Writing – review & editing. YC: Formal analysis, Investigation, Methodology, Resources, Writing – original draft. XW: Formal analysis, Investigation, Methodology, Project administration, Resources, Software, Supervision, Visualization, Writing – original draft, Writing – review & editing.
