# Supplementary material for: Efficacy of MSC-derived small extracellular vesicles in treating type II diabetic cutaneous wounds: a systematic review and meta-analysis of animal models
Source: Front Endocrinol (Lausanne). 2024 Jul 15;15:1375632. doi: 10.3389/fendo.2024.1375632 (PMC11284036; doi:10.3389/fendo.2024.1375632)
Supplement: Supplementary file 1 [file DataSheet_1.docx]

**Table S1.** Search Strategy

Database: Pubmed <2015 to June 14, 2023>, Web of science <2009 to June 14, 2023>, Embase <2016 to June 14, 2023>, Cochran library <2000 to June 14, 2023>

Search Strategy:

**PUBMED**

#1 (Diabetic Foot [MeSH Terms]) OR (Foot, Diabetic[Title/Abstract]) OR (Diabetic Feet[Title/Abstract]) OR (Feet, Diabetic[Title/Abstract]) OR (Foot Ulcer, Diabetic[Title/Abstract]) OR (Healing, Wound[Title/Abstract]) OR (Healings, Wound[Title/Abstract]) OR (Wound Healing[MeSH Terms]) OR (Wound Healings[Title/Abstract]) OR (skin regeneration[Title/Abstract])

#2 (diabetes mellitus[MeSH Terms]) OR (diabet*[Title/Abstract]) OR (T2DM[Title/Abstract]) OR (Hyperglycemia*) OR (Hyperglycemia*, Postprandial) OR (Postprandial Hyperglycemia*)

#3 (Exosomes[MeSH Terms]) OR (microvesicle*[Title/Abstract]) OR (Extracellular Vesicles[MeSH Terms]) OR (Exovesicle*[Title/Abstract]) OR (microparticle*[Title/Abstract]) OR (small Extracellular Vesicle*[Title/Abstract])

#4 (Mesenchymal Stem Cells[MeSH Terms]) OR (Stem Cell*, Mesenchymal[Title/Abstract]) OR (Mesenchymal Stem Cell*[Title/Abstract]) OR (Bone Marrow Mesenchymal Stem Cell*[Title/Abstract]) OR (Bone Marrow Stromal Cell*[Title/Abstract]) OR (Bone Marrow Stromal Cell*, Multipotent[Title/Abstract]) OR (Multipotent Bone Marrow Stromal Cell*[Title/Abstract]) OR (Adipose Derived Mesenchymal Stem Cells*[Title/Abstract]) OR (Adipose-Derived Mesenchymal Stromal Cell*[Title/Abstract]) OR (Adipose Derived Mesenchymal Stromal Cell*[Title/Abstract]) OR (Mesenchymal Stem Cell*, Adipose-Derived[Title/Abstract]) OR (Mesenchymal Stem Cell*, Adipose Derived[Title/Abstract]) OR (Adipose-Derived Mesenchymal Stem Cell*[Title/Abstract]) OR (Adipose Derived Mesenchymal Stem Cell*[Title/Abstract]) OR (Adipose Tissue-Derived Mesenchymal Stem Cell*[Title/Abstract]) OR (Adipose Tissue Derived Mesenchymal Stem Cell*[Title/Abstract]) OR (Adipose Tissue-Derived Mesenchymal Stromal Cell*[Title/Abstract]) OR (Adipose Tissue Derived Mesenchymal Stromal Cell*[Title/Abstract]) OR (Mesenchymal Stromal Cell*[Title/Abstract]) OR (Stromal Cell*, Mesenchymal[Title/Abstract]) OR (Multipotent Mesenchymal Stromal Cell*[Title/Abstract]) OR (Mesenchymal Stromal Cells, Multipotent[Title/Abstract]) OR (Mesenchymal Progenitor Cell*[Title/Abstract]) OR (Progenitor Cell*, Mesenchymal[Title/Abstract]) OR (Wharton's Jelly Cell*[Title/Abstract]) OR (Whartons Jelly Cell*[Title/Abstract]) OR (Bone Marrow Stromal Stem Cell*[Title/Abstract]) OR (Fibroblast[MeSH Terms]) OR (Fibroblast*[Title/Abstract])

#5 #1 AND #2 AND #3 AND #4

**85**

**Web of Science**

#1 TS=(Hyperglycemia*) OR (Hyperglycemia*, Postprandial) OR (Postprandial Hyperglycemia*) OR (diabetes mellitus) OR (diabet*) OR (T2DM)

#2 TS=(Diabetic Foot) OR (Foot, Diabetic) OR (Diabetic Feet) OR (Feet, Diabetic) OR (Foot Ulcer, Diabetic) OR (Healing, Wound) OR (Healings, Wound) OR (Wound Healing) OR (Wound Healings)

#3 TS=(Exosome*) OR (microvesicle*) OR (Extracellular Vesicle*) OR (Exovesicle*) OR (microparticle*) OR (small Extracellular Vesicle*)

#4 TS=(Stem Cell*, Mesenchymal) OR (Mesenchymal Stem Cell*) OR (Bone Marrow Mesenchymal Stem Cell*) OR (Bone Marrow Stromal Cell*) OR (Bone Marrow Stromal Cell*, Multipotent) OR (Multipotent Bone Marrow Stromal Cell*) OR (Adipose Derived Mesenchymal Stem Cells*) OR (Adipose-Derived Mesenchymal Stromal Cell*) OR (Adipose Derived Mesenchymal Stromal Cell*) OR (Mesenchymal Stem Cell*, Adipose-Derived) OR (Mesenchymal Stem Cell*, Adipose Derived) OR (Adipose-Derived Mesenchymal Stem Cell*) OR (Adipose Derived Mesenchymal Stem Cell*) OR (Adipose Tissue-Derived Mesenchymal Stem Cell*) OR (Adipose Tissue Derived Mesenchymal Stem Cell*) OR (Adipose Tissue-Derived Mesenchymal Stromal Cell*) OR (Adipose Tissue Derived Mesenchymal Stromal Cell*) OR (Mesenchymal Stromal Cell*) OR (Stromal Cell*, Mesenchymal) OR (Multipotent Mesenchymal Stromal Cell*) OR (Mesenchymal Stromal Cells, Multipotent) OR (Mesenchymal Progenitor Cell*) OR (Progenitor Cell*, Mesenchymal) OR (Wharton's Jelly Cell*) OR (Whartons Jelly Cell*) OR (Bone Marrow Stromal Stem Cell*) OR (Fibroblast*)

#5 #1 AND #2 AND #3 AND #4

198

**Cochran library:**

#1 MeSH descriptor: [Diabetic Foot] explode all trees

#2 MeSH descriptor: [Wound Healing] explode all trees

#3 ((Foot, Diabetic) OR (Diabetic Feet) OR (Feet, Diabetic) OR (Foot Ulcer, Diabetic) OR (Healing, Wound) OR (Healings, Wound) OR (Wound Healings)):ti,ab,kw

#4 #1 OR #2 OR #3

#5 MeSH descriptor: [Diabetes Mellitus] explode all trees

#6 ((diabet*) OR (T2DM)):ti,ab,kw

#7 #5 OR #6

#8 MeSH descriptor: [Exosomes] explode all trees

#9 MeSH descriptor: [Extracellular Vesicles] explode all trees

#10 ((microvesicle*) OR (Exovesicle*) OR (microparticle*) OR (small Extracellular Vesicle*)):ti,ab,kw

#11 #8 OR #9 OR #10

#12 MeSH descriptor: [Mesenchymal Stem Cells] explode all trees

#13 MeSH descriptor: [Fibroblasts] explode all trees

#14 ((Stem Cell*, Mesenchymal) OR (Mesenchymal Stem Cell*) OR (Bone Marrow Mesenchymal Stem Cell*) OR (Bone Marrow Stromal Cell*) OR (Bone Marrow Stromal Cell*, Multipotent) OR (Multipotent Bone Marrow Stromal Cell*) OR (Adipose Derived Mesenchymal Stem Cells*) OR (Adipose-Derived Mesenchymal Stromal Cell*) OR (Adipose Derived Mesenchymal Stromal Cell*) OR (Mesenchymal Stem Cell*, Adipose-Derived) OR (Mesenchymal Stem Cell*, Adipose Derived) OR (Adipose-Derived Mesenchymal Stem Cell*) OR (Adipose Derived Mesenchymal Stem Cell*) OR (Adipose Tissue-Derived Mesenchymal Stem Cell*) OR (Adipose Tissue Derived Mesenchymal Stem Cell*) OR (Adipose Tissue-Derived Mesenchymal Stromal Cell*) OR (Adipose Tissue Derived Mesenchymal Stromal Cell*) OR (Mesenchymal Stromal Cell*) OR (Stromal Cell*, Mesenchymal) OR (Multipotent Mesenchymal Stromal Cell*) OR (Mesenchymal Stromal Cells, Multipotent) OR (Mesenchymal Progenitor Cell*) OR (Progenitor Cell*, Mesenchymal) OR (Wharton's Jelly Cell*) OR (Whartons Jelly Cell*) OR (Bone Marrow Stromal Stem Cell*) OR (Fibroblast*)):ti,ab,kw

#15 #12 OR #13 OR #14

#16 #4 AND #7 AND #11 AND #15

**0**

**Embase:**

#1 'diabetic foot'/exp OR 'foot, diabetic':ab,ti OR 'diabetic feet':ab,ti OR 'feet, diabetic':ab,ti OR 'foot ulcer, diabetic':ab,ti OR 'healing, wound':ab,ti OR 'healings, wound':ab,ti OR 'wound healing[mesh terms]':ab,ti OR 'wound healings':ab,ti OR 'skin regeneration':ab,ti

#2 'diabetes mellitus'/exp OR 'diabet*':ab,ti OR 't2dm':ab,ti OR hyperglycemia*:ab,ti OR 'hyperglycemia*, postprandial':ab,ti OR 'postprandial hyperglycemia*':ab,ti

#3 'exosomes'/exp OR 'membrane microparticle*':ab,ti OR 'exovesicle*':ab,ti OR 'microparticle*':ab,ti OR 'small extracellular vesicle*':ab,ti OR 'extracellular vesicles'/exp

#4 'mesenchymal stem cells'/exp OR 'stem cell*, mesenchymal':ab,ti OR 'mesenchymal stem cell*':ab,ti OR 'bone marrow mesenchymal stem cell*':ab,ti OR 'bone marrow stromal cell*':ab,ti OR 'bone marrow stromal cell*, multipotent':ab,ti OR 'multipotent bone marrow stromal cell*':ab,ti OR 'adipose derived mesenchymal stem cells*':ab,ti OR 'adipose-derived mesenchymal stromal cell*':ab,ti OR 'adipose derived mesenchymal stromal cell*':ab,ti OR 'mesenchymal stem cell*, adipose-derived':ab,ti OR 'mesenchymal stem cell*, adipose derived':ab,ti OR 'adipose-derived mesenchymal stem cell*':ab,ti OR 'adipose derived mesenchymal stem cell*':ab,ti OR 'adipose tissue-derived mesenchymal stem cell*':ab,ti OR 'adipose tissue derived mesenchymal stem cell*':ab,ti OR 'adipose tissue-derived mesenchymal stromal cell*':ab,ti OR 'adipose tissue derived mesenchymal stromal cell*':ab,ti OR 'mesenchymal stromal cell*':ab,ti OR 'stromal cell*, mesenchymal':ab,ti OR 'multipotent mesenchymal stromal cell*':ab,ti OR 'mesenchymal stromal cells, multipotent':ab,ti OR 'mesenchymal progenitor cell*':ab,ti OR 'progenitor cell*, mesenchymal':ab,ti OR 'whartons jelly cell*':ab,ti OR 'bone marrow stromal stem cell*':ab,ti OR 'fibroblast'/exp OR fibroblast*:ab,ti

#1 AND #2 AND #3 AND #4

**58**

**Table S2.** Egger's test

| **Std_Eff** | **Coef.** | **Std. Err.** | **t** | **P>\|t\|** |
| --- | --- | --- | --- | --- |
| **slope** | **.6549738** | **.3598774** | **1.82** | **0.085** |
| **bias** | **2.426888** | **.3113402** | **7.79** | **0.000** |

**Table S3.** Trim-and-fill

|  | **Pooled** | **95% CI** | | **Asymptotic** | | **No. of studies** |
| --- | --- | --- | --- | --- | --- | --- |
| **Method** | **Est** | **Lower** | **Upper** | **z_value** | **p_value** | **20** |
| **Fixed** | **3.156** | **2.649** | **3.662** | **12.2093.156** | **0.000** |  |
| **Random** | **3.524** | **2.807** | **4.241** | **9.633** | **0.000** |  |

**Trimming estimator: Linear**

**Meta-analysis type: Fixed-effects model**

|  | **Pooled** | **95% CI** | | **Asymptotic** | | **No. of studies** |
| --- | --- | --- | --- | --- | --- | --- |
| **Method** | **Est** | **Lower** | **Upper** | **z_value** | **p_value** | **20** |
| **Fixed** | **15.229** | **9.512** | **24.381** | **11.341** | **0.000** |  |
| **Random** | **17.537** | **8.8086** | **38.033** | **7.252** | **0.000** |  |


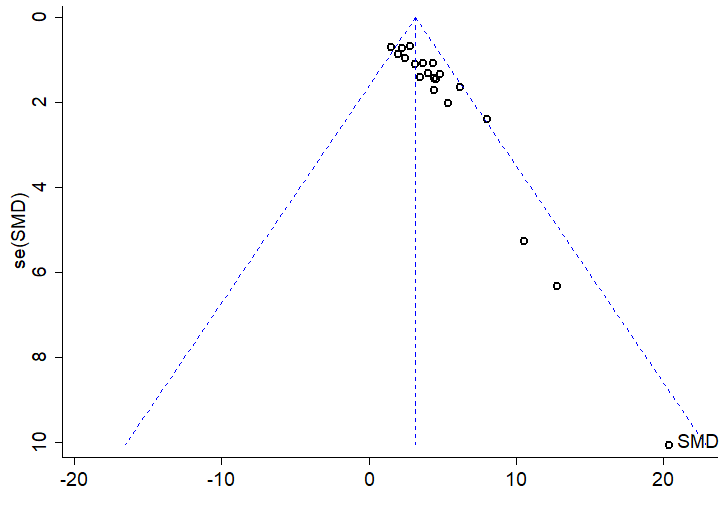


**Figure S1. Funnel Plot analysis assessing publication bias for Wound Closure.**

**
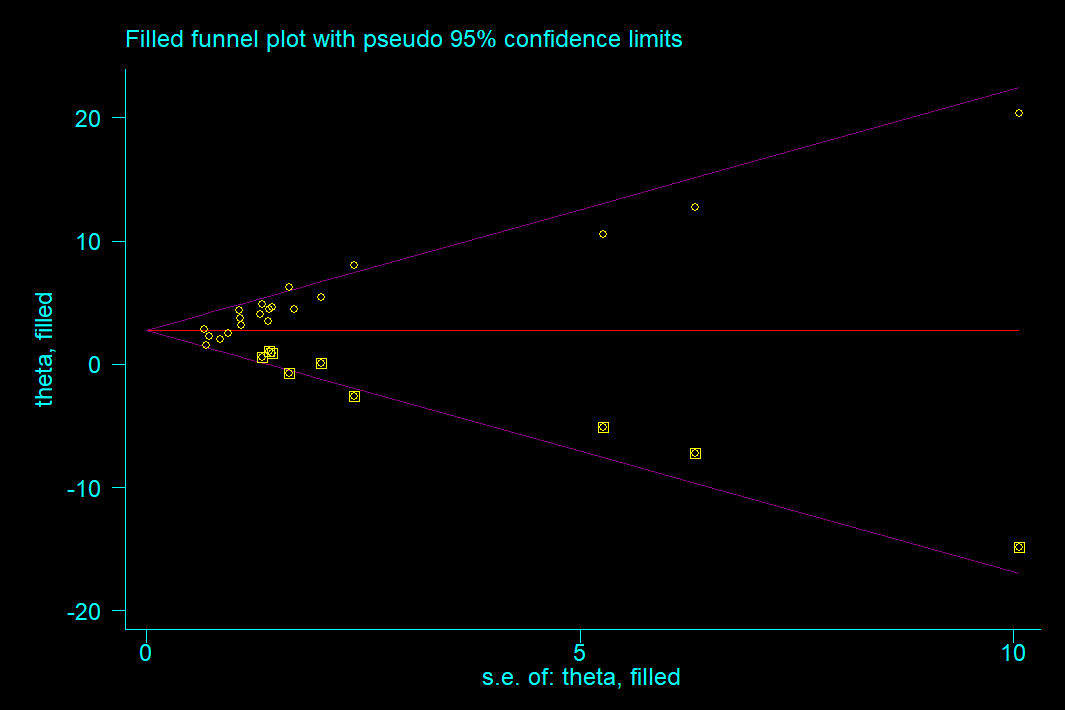
**

**Figure S2. Trim-and-fill method assessing publication bias for Wound Closure.**
